# Knowledge, attitude and practice related to infant feeding among women in rural Papua New Guinea: a descriptive, mixed method study

**DOI:** 10.1186/1746-4358-8-16

**Published:** 2013-11-21

**Authors:** Jerzy Kuzma

**Affiliations:** 1 Faculty of Health Sciences, Divine Word University, PO Box 483, Madang, Papua New Guinea

**Keywords:** Exclusive breastfeeding, Bottle feeding, Infant formula, Infant feeding, Papua New Guinea

## Abstract

**Background:**

Despite the well-recognized effectiveness of exclusive breastfeeding for the first six months of an infant life for reducing infant mortality, adherence to this practice is not widespread in the developing world. Although several studies on infant nutrition practices have been conducted in urban settings of Papua New Guinea (PNG), there is only scant information on infant feeding practices in rural settings. Therefore, this study aimed to investigate knowledge, attitude and practice associated with exclusive breastfeeding in various locations in rural PNG.

**Methods:**

A mixed method study using interviews based on a semi-structured questionnaire (n = 140) and Focus Group Discussions (FGDs) was conducted among mothers in rural PNG between August and September 2012. Participants were selected using convenience sampling. Included in the study were both primiparous and multiparous mothers with a child below the age of two years. Content analysis was used for qualitative data and descriptive statistics were used for quantitative data.

**Results:**

Whereas most women indicated breastfeeding as a better way to feed babies, knowledge of the reasons for its superiority over infant formula was generally poor. Only 17% of mothers practiced exclusive breastfeeding for the first six months postpartum. Our study showed that the size of the gap between exclusive breastfeeding practice and global recommendations was striking. Taking into account the low educational profile of the participants, the disparity may be explained by the fact that most of the mothers in this study had no formal education on infant feeding.

**Conclusions:**

This study showed a lack of understanding of the importance of and poor adherence to exclusive breastfeeding for the first six months postpartum among rural mothers. As exclusive breastfeeding promotion has been proved to be one of most effective ways to improve infant survival, more attention should be given to it, especially targeting the large proportion of women who missed formal education on infant feeding in school. A proper community-based program including the tools for monitoring its implementation and effectiveness needs to be developed to transform policy recommendations into action in rural PNG.

## Background

Promotion of exclusive breastfeeding practices for the first six months of an infant’s life is one of the most effective interventions for reducing infant morbidity and mortality in resource-constrained settings [1]. The World Health Organization (WHO) recommends exclusive breastfeeding for the first six months of life [2,3]. Numerous studies have underlined the advantages of exclusive breastfeeding for growth, immunity and prevention of illness in young infants [4-6]. Conversely, several studies associate lack of exclusive breastfeeding feeding with high infant mortality and morbidity from malnutrition and infections [7,8].

Despite the well-recognized importance of exclusive breastfeeding, this practice is not widespread in the developing world and the increase at the global level is very modest with much room for improvement [9,10]. Infant nutrition programs worldwide continue to require investment and commitment to improve feeding practices in order to have maximum impact on reducing infant morbidity and mortality [10].

Papua New Guinea (PNG) was the first country in the world to introduce legislation to protect breastfeeding with the *Baby Food Supplies* (*Control*) *Act 1977*. This legislation had an immediate impact on the prevalence of infant formula feeding, reducing it in Port Moresby by a third between 1976 and 1979 [10]. However, the impact seemed to be short-lived and a large survey on infant feeding practices in 1995 reported that 20% of mothers in different parts of PNG had used regularly infant formula [11]. Several studies on infant nutrition practices have been conducted in PNG, predominantly in urban settings [11-13]. However, there is little information on infant feeding practices in the rural setting.

This study aims to investigate the knowledge, attitude, practice and social support connected with infant feeding in various locations of rural PNG.

## Methods

This mixed methods study, with convergent parallel design, was conducted between August and September 2012. To explore the issue deeper and inform the interview questionnaire construction, two Focus Group Discussions (FGDs) were undertaken (with 4 and 8 participants respectively) with women not participating in the interviews. The main topics for the FGDs were practice and customs related to breastfeeding.

Interviews in the PNG language Tok Pisin based on a semi-structured questionnaire which included both open-ended and closed questions were used to collect information from mothers (n = 140) in rural locations of the Eastern Highlands and Madang Provinces. Field workers (university students) were trained by the author to conduct the interviews. Convenience sampling was used to select mothers visiting health centres who had a child of less than two years old. Mothers who were currently or previously working as health workers or who were unable to speak Tok Pisin were excluded. Mothers with infants presenting specific feeding problems (cleft palate or lip, severely ill) were also excluded.

The FGDs and all open-ended questions in the interviews were recorded with a voice recorder and transcribed for analysis, whereas closed-ended questions were manually recorded in the questionnaire. The qualitative and quantitative strands of data were analyzed separately and triangulation was performed at the level of inferences. Content analysis was performed for the qualitative data, and main themes were identified according to common threads in the data. The quantitative data were summarized by descriptive statistics using the mean for continuous and proportions for categorical variables.

The study received ethical clearance from the Divine Word University Research Ethics Committee (No1/3/12).

## Results

### Demographic characteristics

In total 140 mothers with an average age of 29 years (range 18–36 years) participated in the questionnaire study. Demographic characteristics of the sample are shown in Table 1.

**Table 1:** Demographic characteristics of the study sample

**Characteristic**	**n (%)**
Ethnicity	
Madang Coastal Province	100 (71)
Eastern Highlands Province (Okapa District)	40 (29)
Parity	
Primipara	76 (54)
Multipara	64 (46)
Maternal age	
< 30 years	85 (61)
≥ 30 years	55 (39)
Maternal education level	
≤ Grade 4	42 (30)
Grade 5–8	62 (44)
> Grade 8	26 (26)
Maternal employment	
Unemployed*	133 (95)
Employed	7 (5)

*mothers have no formal job, they are mostly self-employed subsistence farmers.

### Breastfeeding practice

Ninety eight percent of the mothers (n = 138) were breastfeeding their babies but only two-thirds had given colostrum to their babies (n = 96). Most explanations given by mothers for refusing to feed their babies colostrum were connected with cultural beliefs that “colostrum may harm the baby”. Colostrum was described as “dirty”, “unclean”, “contained infected pus”, “waste from mother’s body”, “not food for the baby”, and “infectious to a child and can cause yellow eyes”.

Ninety one percent (n = 128) of mothers breastfed their babies when they cried, which amounted to 7–8 times per day. Eighty three percent (n = 116) of interviewed mothers supported their babies under six months with additional food. Interestingly, supplementary feeding of babies under six months was more common (88%) among older (≥ 30 years of age) than younger mothers (80%), however this difference was not significant (p = 0.23; Pearson Chi-square test). In the FGDs all mothers admitted starting supplementary food for their babies at three to four months of age. These included semi-solid food, such as mashed sweet potatoes, papaya, potatoes, banana, pumpkin and soup. Common explanations for early supplementary feeding of their babies were that supplementary food “helps the child to grow well” or that “breast milk is not sufficient for the child to grow and be strong”.

More than a half of the mothers (58%, n = 81) stopped breastfeeding within 12 months. Of note is that a greater proportion of older mothers (78%; 40/55) compared to younger mothers (35%; 41/85) were still breastfeeding when their baby was 12 months of age (p = 0.06; Pearson Chi-square test). Factors that influenced the breastfeeding period were: new pregnancy, work demands (gardening, marketing, housework, and professional careers), child choice, needing to go back to their studies, and family problems. Almost two-thirds of mothers (66%, n = 92) experienced good family support during their breastfeeding period. Qualitative data from the FGDs and open-ended questions in the interview confirmed the quantitative findings.

### Attitude towards breastfeeding

The majority of mothers (87.9%, n = 123) regarded breastfeeding as good, giving various reasons for their perception relating to this (Table 2).

**Table 2:** Attitudes of lactating mothers towards breastfeeding

Reason*	**%**	**n**
Healthy for the child	48	67
Helps the child to grow well	38	53
Breast milk is the best food for the child	36	51
Beneficial to the mother (reduces breast tightness and pain)	63	88
Bad for the mother (causes weight loss)	17	22
No known benefit	23	32

*many gave more than one reason.

Less common explanations given in support of breastfeeding were: “it is in line with the culture”, “mother shows responsibility for a baby”, “breastfeeding is easy – no need to prepare food for a baby”, and some believed that it prevents pregnancy.

According to mothers, most husbands (99%, n = 138) were happy and supportive towards breastfeeding, however 27% (n = 39) said that their husbands had influenced them to shorten the period of breastfeeding. A frequently given explanation for why husbands would have influenced mothers to shorten breastfeeding was a custom that did not allow having sex with a breastfeeding woman. This is a good illustration where the qualitative strand of data provided explanations for the quantitative data.

### Food taboos/restriction

More than half the lactating mothers (57%; n = 80) admitted that food restrictions were imposed on them by their culture. In particular a higher proportion of older (80%) than younger mothers (42%) admitted food restrictions during lactation. Those restrictions vary in different areas and include fish from the sea, pork fat, pork meat, possum (cuscus), eels, red pandamus fruit (*Pandamus conoideus*), sugar cane, short banana and taro. Breaking those taboos was believed to cause the child to become sick or crippled, stunted in growth or attain undesirable features of the eaten animal. For instance a mother from the Madang Coast believed that, “if a lactating woman eats bandicoot, her baby would develop tremor and become sick”. Another breastfeeding mother stated, “I am not allowed to eat octopus because my baby may develop shortness of breath.”

### Infant formula feeding practice

Even though the large proportion of mothers (77%, n = 108) considered infant formula feeding as not good, knowledge about its disadvantages was very low. One third of participants (30%, n = 42) did not know why infant formula feeding was not good. Interestingly, all mothers lacking knowledge of the hazards associated with infant formula feeding were below Grade 8 educational level. Although 70% of mothers (n = 98) considered that infant formula feeding was not healthy and some noted that it was expensive, very few thought it could cause diarrhoea or illness.

## Discussion

### Practice of infant feeding

A global evidence-based public health resolution recommends exclusive breastfeeding for the first six months of life and continued breastfeeding up to two years of age and beyond [14]. In low-resource countries the prevalence of exclusive breastfeeding at six months is generally low and varies from 9% [15] to 39% [16].

Although this study showed that almost all rural mothers (98%) breastfed their babies, exclusive breastfeeding up to six months of baby’s life was not commonly understood and was practiced by only 17% of mothers. This is consistent with other low-resource countries. The prevalence of breastfeeding in our study differed considerably from findings of a recent study in PNG where 40% of mothers in urban setting had an experience with infant formula feeding at some stage of child’s life [13]. Differences in the employment status of mothers in rural (5% employed) and urban (30% employed) might partially account for the difference.

In our study the prevalence of continued breastfeeding for infants and children aged 12–23 months was average for a low-resource country [2]. The sizable gap between breastfeeding practice in developing countries and international recommendations indicates that more attention should be given to the promotion of exclusive breastfeeding. Breastfeeding promotion interventions in developing countries have been shown to result in a six-fold increase in exclusive breastfeeding rates at six months [17]. Furthermore, a recent review of child survival interventions in low-income countries showed that the promotion of breastfeeding was one of the most effective public health interventions, reducing under-five mortality by 13% [1]. Breastfeeding promotion, therefore, has become a global health priority with maximum impact on both maternal and child health in developing countries.

The HIV epidemic has added another aspect to the importance of best infant feeding practice. For HIV-positive mothers, exclusive breastfeeding for the first six months of a baby’s life offers the lowest risk of transmission and child mortality, whereas suboptimal (mixed) feeding is associated with a higher transmission and child mortality rate [18].

### Knowledge and attitude towards breastfeeding

Even though most rural mothers regarded breastfeeding as the best for their babies, knowledge about the benefits of breastfeeding and hazards of infant formula feeding was very low. Taking into account the low educational profile of the participants and the fact that in the current curriculum infant feeding is supposedly to be taught in Grade 8, this may be explained by the fact that many of the mothers in this study may have missed that formal education on infant feeding.

According to the participants, almost all fathers (99%) supported breastfeeding; however a quarter of fathers influenced the earlier stopping of breastfeeding. This may be explained by a common belief in PNG that a couple should not resume sexual relations whilst a woman is breastfeeding. A Vietnamese study also found that the husband and maternal mother influenced breastfeeding practice [19]. The majority of the mothers in this study experienced family support for breastfeeding. Another study indicated that peer support increased the duration of exclusive breastfeeding in low and middle-income countries [20].

In this study traditional and customary beliefs influenced infant feeding practice in two areas: feeding with colostrum and imposing various dietary restrictions on lactating women. This finding is consistent with a study of breastfeeding practices in India [21]. Food taboos are known in various cultures and are designed to protect humans from real or assumed health hazards [22]. A better understanding of customary beliefs towards lactating women will allow more effective breastfeeding education and promotion.

### Limitations of the study

There were several limitations in our study. Non-probability sampling with likely sampling bias, a relatively small sample size and rural locations in only two provinces together with the rich cultural diversity in rural PNG mean that our results cannot be fully extrapolated to the whole rural population.

## Conclusions and recommendations

Rural mothers’ knowledge about the benefits of breastfeeding and in particular about the importance of exclusive breastfeeding in a child’s first six months of life is poor. This study showed that the size of the gap between exclusive breastfeeding practice in PNG and WHO recommendations is striking.

As exclusive breastfeeding promotions improve infant survival, more attention in health planning should be given to its promotion. In particular, breastfeeding promotion should target the large proportion of women who have missed formal education about infant feeding in school. Greater emphasis should be made at children’s and antenatal clinics to educate mothers about the importance of exclusive breastfeeding for the first six months of their infants’ life, the advantages of breastfeeding, and potential hazards of feeding a baby with infant formula. Although the PNG National Health Plan 2011–2020 states that to improve child survival, health workers must place more emphasis on exclusive breastfeeding [23], proper planning, including community-based programs and the tools for monitoring its implementation and effectiveness are yet to be developed.

## Consent

Written informed consent was obtained from all participants of the study for the publication of this report.

## Competing interests

The author declares that they have no competing interests.
